# The Neuroprotective Effect of Astaxanthin on Pilocarpine-Induced Status Epilepticus in Rats

**DOI:** 10.3389/fncel.2019.00123

**Published:** 2019-03-29

**Authors:** Xiaolin Deng, Ming Wang, Sihui Hu, Yonghao Feng, Yiye Shao, Yangmei Xie, Men Wu, Yinghui Chen, Xiaohong Shi

**Affiliations:** ^1^Department of Neurology, Jinshan Hospital, Fudan University, Shanghai, China; ^2^Department of Neurology, Huashan Hospital North, Fudan University, Shanghai, China; ^3^Department of Endocrinology, Jinshan Hospital, Fudan University, Shanghai, China

**Keywords:** astaxanthin, neuroprotective effect, status epilepticus, oxidative stress, neuroinflammation, apoptosis

## Abstract

Cognitive dysfunction is one of the serious complications induced by status epilepticus (SE), which has a significant negative impact on patients’ quality of life. Previous studies demonstrated that the pathophysiological changes after SE such as oxidative stress, inflammatory reaction contribute to neuronal damage. A recent study indicated that preventive astaxanthin (AST) alleviated epilepsy-induced oxidative stress and neuronal apoptosis in the brain. In the present study, rats were treated with vehicle or AST 1 h after SE onset and were injected once every other day for 2 weeks (total of seven times). The results showed that the cognitive function in SE rats was significantly impaired, and AST treatment improved cognitive function in the Morris water maze (MWM). Magnetic resonance imaging (MRI), hematoxylin-eosin (HE) staining and TdT-mediated dUTP Nick-End Labeling (TUNEL) staining showed obvious damage in the hippocampus of SE rats, and AST alleviated the damage. Subsequently, we evaluated the effect of AST on relative pathophysiology to elucidate the possible mechanisms. To evaluate the oxidative stress, the expression of malondialdehyde (MDA) and superoxide dismutase (SOD) in plasma were detected using commercially available kits. NADPH oxidase-4 (Nox-4), p22phox, NF-E2-related factor 2 (Nrf-2), heme oxygenase 1 (Ho-1) and sod1 in the parahippocampal cortex and hippocampus were detected using western blot and real-time polymerase chain reaction (RT-PCR). The levels of MDA in plasma and Nox-4 and p22phox in the brain increased in SE rats, and the levels of SOD in plasma and Nrf-2, Ho-1 and sod1 in the brain decreased. Treatment with AST alleviated these changes. We also detected the levels of inflammatory mediators like cyclooxygenase-2 (cox-2), interleukin-1β (IL-1β), tumor necrosis factor-α (TNF-α) and NF-κB phosphorylation p65 (p-p65)/p65 in the brain. The inflammatory reaction was significantly activated in the brain of SE rats, and AST alleviated neuroinflammation. We detected the levels of p-Akt, Akt, B-cell lymphoma-2 (Bcl-2), Bax, cleaved caspase-3, and caspase-3 in the parahippocampal cortex and hippocampus using western blot. The levels of p-Akt/Akt and Bcl-2 decreased in SE rats, Bax and cleaved caspase-3/caspase-3 increased, while AST alleviated these changes. The present study indicated that AST exerted an reobvious neuroprotective effect in pilocarpine-induced SE rats.

## Introduction

Status epilepticus (SE) is one of the most common neurological emergency diseases, which is ascribed to the failure of the mechanisms responsible for seizure termination or the initiation of mechanisms leading to abnormally prolonged seizures. Accumulating evidence demonstrated that SE results in extensive brain damage or death (Trinka et al., 2015). SE exhibits an incidence of 10 per 100,000 persons per year to 41 per 100,000 persons per year, and it has short-term total mortality rates of approximately 20%, of which 45%–74% are generalized convulsive SE. Various factors cause the occurrence of SE, including multiple neurological diseases, such as stroke, brain tumor, brain trauma, central nervous system infection and autoimmune encephalitis, and inappropriate medications during the course of epilepsy treatment. The prognosis of SE is poor, which often results in serious consequences, ranging from transient neurological dysfunction to life-threatening issues if not competently managed, including irreversible brain damage. The longer SE lasts, the worse the consequences (Betjemann and Lowenstein, 2015; Trinka et al., 2016; Yasam et al., 2017; Sculier et al., 2018).

The present treatment of SE is based on a primary principle, termination of the seizure as soon as possible. Treatment drugs include benzodiazepines, antiepileptic drugs (AEDs) and some anesthetics, which control approximately two-thirds of seizures. However, these drugs often exacerbate comorbidities, such as cognitive impairment (Agarwal et al., 2011; Sutter et al., 2014). Post-epileptic neuronal injury is widely recognized, but the underlying mechanism is still unclear. Currently, there is a lack of effective treatment for post-SE neuronal injury. The pathophysiology underlying SE includes neuroinflammation, oxidative stress, neuronal death and abnormal neuron networks, which aggravate epileptic etiological changes and neuronal damage after SE, thus inducing cognitive impairment. Current AEDs can terminate most seizures, but the above-mentioned early pathophysiological changes induced by SE are not fully inhibited by AEDs. These changes promote the occurrence of seizure, which results in a recurrent seizure, and thus developing into chronic temporal lobe epilepsy (TLE; Pitkänen, 2010; Pitkänen and Lukasiuk, 2011; Pitkänen et al., 2019).

Astaxanthin (AST) is a lutein carotenoid, which is widely found in a variety of micro- and marine organisms, such as algae, yeast, salmon, trout, krill, shrimp and crayfish. Previous studies demonstrated that AST was a powerful antioxidant because of its peculiar molecular structure of a conjugated double bond at the center and unsaturated ketonic groups at both ends of the aromatic nucleus (Ambati et al., 2014). Previous studies indicated that AST directly alleviated oxidative stress and indirectly oxidative damage *via* enhancing the activity of the brain NF-E2-related factor 2 (Nrf-2)/anti-oxidant response element (ARE) antioxidant pathway (Grimmig et al., 2017). Additionally, AST exhibits multipotent biological characteristics, such as anti-inflammation, anti-apoptosis, anti-tumor and immunity enhancement (Ying et al., 2015; Kim et al., 2017). AST easily crosses the blood-brain barrier, and it has no toxic side effects (Ambati et al., 2014). AST reduced brain damage and ameliorated cognitive impairment in some neurological disorders (Ji et al., 2017). One study showed that preventive AST prior to epilepsy induced by amygdala kindling inhibited neuron loss and oxidative stress (Lu et al., 2015). However, the efficacy of AST treatment administration after a complete SE is unknown. Therefore, we evaluated the effects of AST treatment beginning 1 h after SE for 2 weeks on the cognitive function of post-SE rats, hippocampal damage, oxidative stress, inflammation and neuronal loss using the Morris water maze (MWM), magnetic resonance imaging (MRI), hematoxylin-eosin (HE) staining, TdT-mediated dUTP Nick-End Labeling (TUNEL) staining, western blot and real-time polymerase chain reaction (RT-PCR).

## Materials and Methods

### Animals

Male Wistar rats (*n* = 104; body weight 160–180 g) were used in the study and were purchased from the Animal Center of Fudan University (Shanghai, China). All rats were raised in a standard animal-grade room with four or five rats in each cage. This study was carried out in accordance with the recommendations of Guide for Animal Experiments of the Chinese Academy of Medical Sciences. The protocol was approved by the ethics committee for animal care of Jinshan Hospital of Fudan University. The housing environment met the following conditions: ambient temperature approximately 23 ± 2°C, relative humidity (60 ± 5%) and a 12 h light/dark cycle with free access to food and water. All animal experiments were performed during the light cycle. All rats were housed in the laboratory for 1 week prior to experimentation.

### Establishment of the Model of Status Epilepticus in Rats

**Figure 1:**
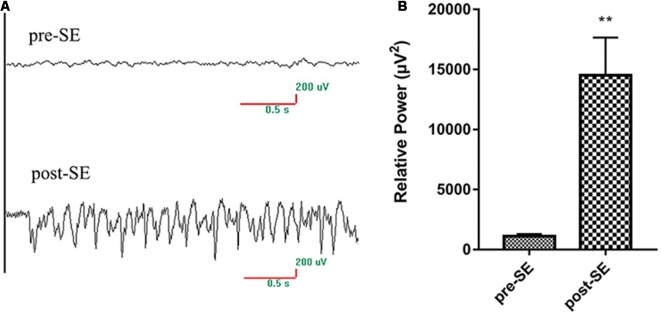
EEG changes of rats pro-SE and post-SE.**(A)** The EEG of rats pro-SE and post-SE; **(B)** the EEG relative power of rats pro-SE and post-SE.The EEG relative power (μV^2^) of SE rats was significantly increased (**p < 0.01 vs. Normal).

Lithium chloride (Sigma, USA) and pilocarpine (Sigma, USA) were used to induce SE (García-García et al., 2017). Rats were intraperitoneally injected with 127 mg/kg lithium chloride in 0.9% saline 20–24 h before pilocarpine injection. Scopolamine (1 mg/kg in 0.9% saline) was injected intraperitoneally 30 min before pilocarpine injection to reduce peripheral side effects. Intraperitoneal injection of 30 mg/kg pilocarpine was performed in the SE and AST groups, and saline was injected into the Normal group as a replacement for lithium chloride and pilocarpine. Electroencephalogram (EEG) monitoring was performed on rats during SE to identify a seizure (Figure 1), and behavioral seizures were scored using a modified Racine scale (Ishida et al., 1992). Seizures were graded using the following classification: I—standing still or wet dog shakes; II—nodding and chewing rhythmically; III—unilateral forelimb clonic seizure; IV—bilateral forelimb clonic and convulsive seizure with standing; and V—receding, tumbling, with a generalized tonic-clonic seizure. SE models were regarded as successfully kindled when the rats developed seizure scoring grade IV-V within 30 min and exhibited a sustained state. If the rat did not develop seizure scoring grade IV-V, then rats were injected (i.p.) with 10 mg/kg pilocarpine every 30 min until the seizure reached grade IV-V. No rat received more than 60 mg/kg pilocarpine. Diazepam (10 mg/kg, i.p.) was administered after a seizure lasted 60 min to terminate the seizure. The criteria for SE model success included EEG showing seizure, seizure grade reaching IV-V and seizure lasting for over 30 min. A total of 104 rats were used. Twenty-two rats were injected with saline for the Normal group, and 82 rats were injected with pilocarpine to induce SE. Sixty-three rats were successfully induced SE rats (success rate 76.83%), and these rats were randomly divided into an SE group (31 rats) and AST group (32 rats). Nine rats failed (failure rate 10.98%), and ten rats died (mortality rate 12.20%).

### Animal Grouping and Experimental Protocol

**Figure 2:**
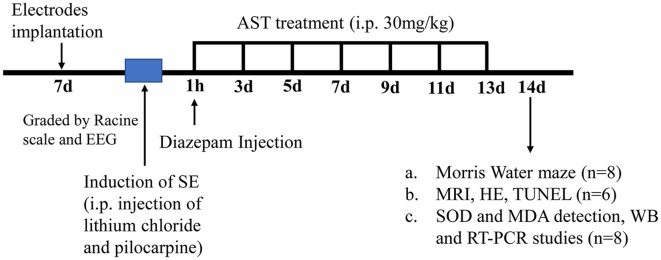
A flow diagram showing the experimental design and the time-line of astaxanthin (AST) treatment. The induction of status epilepticus (SE) was induced in Wistar rats 7 days after electrode implantation. The assessment of successful SE model was using a modified Racine scale and electroencephalogram (EEG) monitoring. Rats received the first injection of 30 mg/kg AST at an hour after the onset of SE, and seizures were terminated *via* i.p. injection of diazepam (10 mg/kg) at the same time. In the following 12 days (days 2–13), rats received injections of AST every other day (total six times). In the 14th day after SE induction, subgroups of rats (*n* = 8) treated with AST started performing Morris Water Maze (MWM) experiment along with SE rats and normal rats; part of rats (*n* = 6) conducted magnetic resonance imaging (MRI), and then euthanized *via* intracardiac perfusions for hematoxylin-eosin (HE) staining and TdT-mediated dUTP Nick-End Labeling (TUNEL) staining; the rest of rats (*n* = 8) were euthanized for biochemical and molecular biological studies [superoxide dismutase (SOD) and malondialdehyde (MDA) detection, WB and real-time polymerase chain reaction (RT-PCR) studies].

Experimental animals were divided into three groups: Normal group, SE group and AST group. Treatments were initiated 60 min after the onset of SE to allow enough time in SE to produce significant physiopathological changes, such as oxidative stress, neuroinflammation, and significant neuron loss, which promote epilepsy development in all rats. The interventions were administered at the same time every 2 days for 2 weeks (total of seven times). AST (purity ≥ 98%; Solarbio, Beijing, China) was dissolved in vehicle mixing 1:1 polyethylene glycol (Sigma, USA) and tri-distilled water. The AST group was intraperitoneally injected with 30 mg/kg AST (Solarbio, Beijing, China), according to previous studies (Lu et al., 2015). The Normal and SE groups received the same dose of vehicle (see Figure 2).

### Electroencephalogram (EEG)

Stainless steel screws for EEG recording were implanted stereotactically on the left and right epidural surfaces [coordinates from the Bregma: anterior-posterior (AP) = −2.2 mm, lateral (L) = ±3.2 mm] under anesthesia with 10% chloral hydrate (Shenggong, Shanghai, China). Two other stainless steel screws were implanted in cerebellar regions as a grounding electrode and reference electrode. The screws were fixed with dental cement (Shanghai, China). Modeling began 7 days after electrode implantation.

### Magnetic Resonance Imaging (MRI)

MRI was performed under anesthesia with 10% chloral hydrate (Shenggong, Shanghai, China). We collected high-resolution T2-weighted phase (T2WI) images of the hippocampus. RARE sequence was used to detect the MRI of the hippocampus of rats using the following parameters: repeat time (TR): 6,000 ms; echo time (TE): 13.7 ms; matrix size: 152 × 152; magnetic resonance FOV: 26 × 26 mm; MRI scanning layer thickness: 0.5 mm; and acquisition time: 3 min 15 s.

### Morris Water Maze Experiment

The MWM consisted of a circular pool in which rats were trained to escape from the water by swimming to a hidden platform, as described previously (Vorhees and Williams, 2006). Adaptive swimming training was performed on the 14th day after modeling. The formal test was performed on the 15th day. Water temperature was maintained at 25 ± 1°C with the addition of warm water. Concealed platform test: the test training stage lasted for 4 days, and rats were trained four times each day. Rats were placed into the pool from four entry points facing the pool wall on the trial each day. The time required for the rats from entering the water to finding the submerged platform and standing on it was recorded as the escape latency (s). When the rats found the platform, they stood on it for 15 s. If the rats failed to find the platform 60 s after entering the water, they were gently dragged onto the platform from the water and stayed in place for 15 s before being removed from the pool. All tests were performed at roughly the same time of day to minimize changes due to time. Spatial probe test: the platform was removed 24 h after the concealed platform test, and the rats were placed into the water at any same entry point one time. The swimming paths within 60 s, the time spent in the target quadrant and the times the rat swam across the original platform site were recorded to evaluate spatial positioning ability.

### HE Staining

The brain tissues of rats were fixed with 4% paraformaldehyde (Shenggong, Shanghai, China), washed, dehydrated, transparentized, immersed in wax, and cut into slices. The dried sections were immersed in a dyeing vessel containing xylene I and xylene II for dewaxing. Sections were successively immersed in different concentrations of alcohol, double-distilled water to hydrate, stained in HE, dehydrated, and sealed. The prepared tissue sections were observed under an optical microscope and photographed using a microimaging system.

### TUNEL Staining

The brain tissues of rats were fixed with 4% paraformaldehyde (Shenggong, Shanghai, China), washed, dehydrated, transparentized, immersed in wax, and cut into slices. The dried sections were immersed in a dyeing vessel containing xylene I and xylene II for dewaxing. The sections were hydrated, digested with trypsin, and stained in TUNEL mix. The sections were dehydrated, transparentized and sealed with neutral gum. The CA1 region of the hippocampus was observed and analyzed using an optical microscope to calculate the apoptosis rate.

### Malondialdehyde (MDA) and Superoxide Dismutase (SOD) Assays

Assays of the malondialdehyde (MDA) content and superoxide dismutase (SOD) activity involved commercially available kits (Jiancheng Bioengineering, Nanjing, China). Plasma was diluted with saline to an appropriate concentration to estimate the MDA content and SOD activity. All procedures were performed under the guidance of the manufacturer’s instructions. MDA levels were measured using a 2-thiobarbital acid method. Optical density was measured at 532 nm. The results were expressed as nmol/mg protein. SOD activity was determined based on its ability to inhibit the oxidation of superoxide anions produced by a xanthine-xanthine oxidase system. One unit of SOD activity was defined as a 50% reduction of optical density at 450 nm absorbance. The results were expressed as U/ml. Colorimetry was used for determination, and the absorbance values and standard equation were used for the assay.

### Western Blot

Hippocampal and cortical tissue proteins were extracted using a lysis buffer containing 1% PMSF (phenyl sulfonyl fluoride; Beyotime, Shanghai, China). Protein content was determined using the BCA assay (BioRad, Hercules, CA, USA). Proteins (20 μg) were separated using SDS-PAGE electrophoresis and transferred to PVDF membranes. The membranes were blocked with 5% BSA/TBST or skim milk at room temperature for 1 h. The membranes were washed with TBST and incubated with a relatively specific primary antibody (Table 1) at 4°C overnight. The membranes were washed with TBST and incubated with horseradish peroxidase (HRP)-conjugated anti-rabbit/mouse IgG at room temperature for 1 h. The ECL-Plus kit (Merck Millipore, Darmstadt, Germany) was used to detect signals, and ImageJ was used to quantify western blots. β-actin was used as an internal control for quantitative analysis of relative expression levels of proteins.

**Table 1:** The specific primary antibodies used in the current study.

Antibody	Company	Dilution
anti-β-actin	Cell Signaling Technology, USA	1:5,000
Nox-4	ABclonal, UK	1:1,000
p22phox	Santa Cruz Biotechnology, USA	1:50
sod1	Proteintech, USA	1:1,000
Ho-1	Abcam, UK	1:1,000
Nrf-2	Santa Cruz Biotechnology, USA	1:400
Cox-2	Cell Signaling Technology, USA	1:500
TNF-α	Millipore, USA	1:1,000
IL-1β	Abcam, UK	1:1,000
p-p65	Cell Signaling Technology, USA	1:1,000
p65	Cell Signaling Technology, USA	1:3,000
p-Akt	Cell Signaling Technology, USA	1:2,000
Akt	Cell Signaling Technology, USA	1:3,000
Bcl-2	Cell Signaling Technology, USA	1:2,000
Bax	Cell Signaling Technology, USA	1:2,000
cleaved caspase-3	Cell Signaling Technology, USA	1:1,000
caspase-3	Cell Signaling Technology, USA	1:3,000
HRP-conjugated anti-rabbit IgG	Proteintech, USA	1:5,000
HRP-conjugated anti-mouse-IgG	Proteintech, USA	1:5,000

### Real-Time Polymerase Chain Reaction (RT-PCR)

The total RNA of hippocampal and cortical brain tissue was extracted using the Trizol Reagent kit (Takara, Japan). All procedures were performed under the guidance of the manufacturer’s instructions. cDNA of the mRNA of target genes was synthesized using the PrimeScript^TM^ reagent kit (Takara, Japan). Primer Express software v3.0.1 was used to design the RT-PCR primers. SYBR Premix Ex Taq (Tli RNaseH Plus; TaKaRa) was used to perform RT-PCR in the Applied Biosystem 7300 (Applied Biosystems, Foster City, CA, USA), to detect the levels of β-actin and target genes. The 2^−ΔΔCt^ method was used to assess the levels of relative mRNA normalized to β-actin. The primer sequences were shown in Table 2.

**Table 2:** The sequences of primers used in the current study.

Gene	Primer	Sequence (5′ to 3′)
Nox-4	Nox-4 (forward)	ACTCTACTGGATGACTGGAAACC
	Nox-4 (reverse)	AGCTGGATGTTCACAAAGTCAGG
p22phox	p22phox (forward)	TGGCGGGCGTGTTTGTGT
	p22phox (reverse)	CCACGGCGGTCATGTACTTC
Ho-1	Ho-1 (forward)	TATCGTGCTCGCATGAACACTCTG
	Ho-1 (reverse)	GTTGAGCAGGAAGGCGGTCTTAG
Nrf-2	Nrf-2 (forward)	GCCTTCCTCTGCTGCCATTAGTC
	Nrf-2 (reverse)	TCATTGAACTCCACCGTGCCTTC
Cox-2	Cox-2 (forward)	CGGACTTGCTCACTTTGTTG
	Cox-2 (reverse)	CTCTCTGCTCTGGTCAATGG
TNF-α	TNF-α (forward)	GCATGATCCGAGATGTGGAACTGG
	TNF-α (reverse)	CGCCACGAGCAGGAATGAGAAG
IL-1β	IL-1β (forward)	GCCAACAAGTGGTATTCTCCA
	IL-1β (reverse)	TGCCGTCTTTCATCACACAG
β-actin	β-actin (forward)	CCACCATGTACCCAGGCATT
	β-actin (reverse)	CAGTGAGGCCAGGATAGAGC

### Statistical Analysis

The results were expressed as the mean value ± SD. All data analyses were performed using GraphPad Prism 7. The paired *t*-test was used to compare the differences between the two groups. The escape latency of the MWM was analyzed using repeated measures ANOVA, with factors of strain and days. *P* < 0.05 was used to indicate significant differences. All experiments were repeated at least three times.

## Results

### Result 1. Astaxanthin Treatment Significantly Improved the Cognitive Function of SE Rats

**Figure 3:**
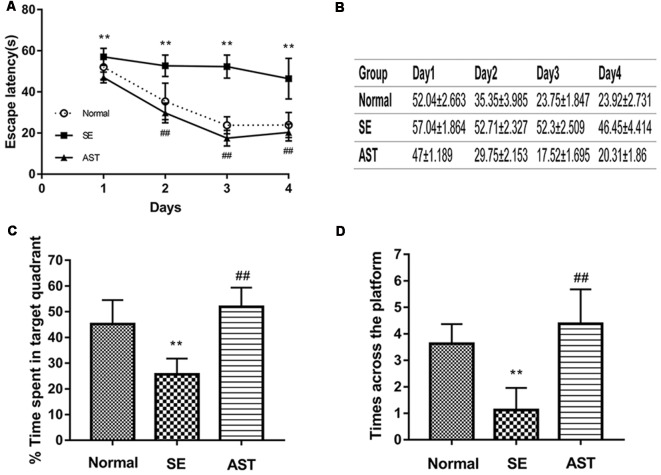
The results of MWM. **(A,B)** Under treatment with 30 mg/kg AST significantly reduces the escape latency. **(C)** The bar chart shows the time spent in target quadrant (%). AST treatment clearly increases the time spent in target quadrant. **(D)** The times across the platform (**p < 0.01 vs. Normal; ^##^*p* < 0.01 vs. SE).

The results indicated that AST significantly ameliorated SE-induced cognitive impairment. The hidden platform test showed that the escape latency in the SE group was longer than the Normal group (**p < 0.01 vs. Normal). However, the escape latency in the AST group was reduced compared to the SE group (^##^*p* < 0.01 vs. SE; Figures 3A,B). The spatial probe test showed that the time spent in the target quadrant and the time across the platform in the SE group were significantly reduced compared to the Normal group, and treatment with AST obviously reversed these changes (**p < 0.01 vs. Normal; ^##^*p* < 0.01 vs. SE; Figures 3C,D). The results of the MWM indicated that SE significantly impaired cognitive function, which is consistent with previous reports (Müller et al., 2009). Treatment with AST ameliorated the SE-induced cognitive impairment.

### Result 2. Astaxanthin Alleviated the SE-Induced Hippocampal Damage

**Figure 4:**
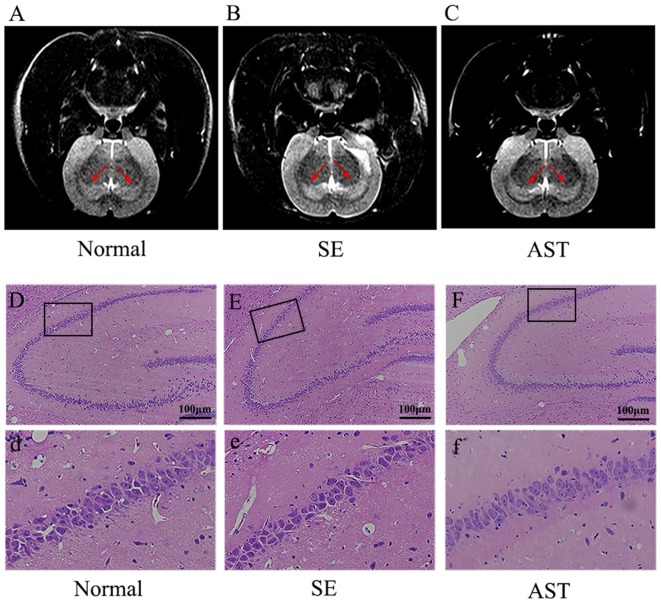
Observation of the hippocampal damage *via* MRI and HE staining. Panels **(A,B)** show the results of T2-weighted phase (T2WI) of MRI. **(A)** Normal group. **(B)** SE group. **(C)** AST group. The T2WIs of the hippocampus of the SE group exhibited a higher signal compared to the Normal group. AST treatment showed a lower signal compared to the SE group. Panels **(D–F)** show the results of HE staining in the hippocampus. **(D,d)** Normal group. **(E,e)** SE group. **(F,f)** AST group. AST treatment improved the morphological changes in SE rats.

The present study showed that AST intervention ameliorated SE-induced hippocampal damage as detected on high-resolution T2WI images of the hippocampus using MRI. As shown in Figures 4A–C, the T2WIs of the hippocampus of the SE group exhibited a higher signal compared to the Normal group. The AST group showed a lower signal compared to the SE group. Our study indicated that SE induced hippocampal damage in rats, such as edema, and that AST intervention alleviated the hippocampal damage.

Meanwhile, we utilized H&E staining to evaluate morphological changes in the rat hippocampus. Neurons in the hippocampus of the SE group were decreased, swollen and loosely arranged compared to the Normal group, and karyopyknosis was obviously observed in the CA1 region of the hippocampus in the SE group (Figures 4Dd,Ee). These morphological dysfunctions were ameliorated in the AST group compared to the SE group (Figure 4Ff).

### Result 3. Astaxanthin Ameliorated Apoptosis of Hippocampal Neurons in SE Rats

**Figure 5:**
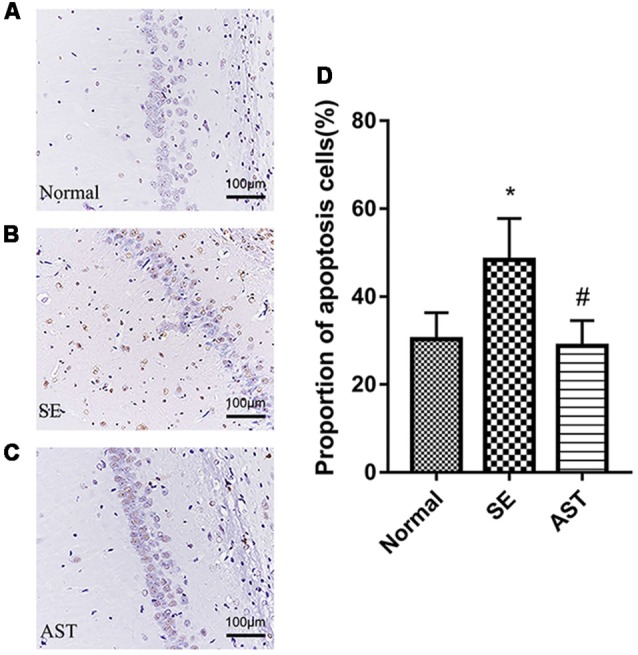
The apoptosis levels in the hippocampal CA1 region *via* TUNEL staining. **(A)** Normal group. **(B)** SE group. **(C)** AST group. **(D)** The bar chart shows a proportion of apoptosis cells in the CA1 region in the hippocampus. The results are expressed as the mean ± SD (*p < 0.05 vs. Normal; ^#^*p* < 0.05 vs. SE).

TUNEL staining showed that AST intervention ameliorated SE-induced apoptosis of hippocampal neurons, and AST intervention ameliorated the apoptosis of neurons in the CA1 region of the hippocampus. As shown in Figure 5, the apoptosis rate of neurons in hippocampus CA1 significantly increased in the SE group (48.36 ± 5.436%) compared to the Normal group (30.3 ± 3.485%; *p* < 0.05). In contrast, AST reduced the apoptosis rate (28.79 ± 3.32%; *p* < 0.05).

### Result 4. Astaxanthin Obviously Reduced Oxidative Stress Status in SE Rats

**Figure 6:**
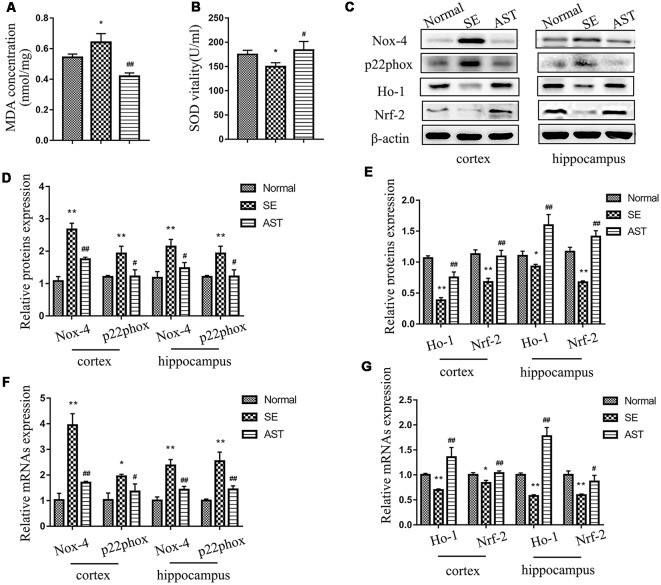
The levels of oxidative stress in each group. **(A)** MDA level in plasma. **(B)** SOD vitality in plasma. **(C)** Representative protein bands of NADPH oxidase-4 (Nox-4), p22phox, NF-E2-related factor 2 (Nrf2), heme oxygenase 1 (HO-1) and sod1 in the parahippocampal cortex and hippocampus. **(D)** The bar chart shows the relative proteins levels of Nox-4 and p22phox in the parahippocampal cortex and hippocampus, compared with that of β-actin. **(E)** The bar chart shows the relative proteins levels of Nrf-2, HO-1 and sod1 in the parahippocampal cortex and hippocampus, compared with that of β-actin. **(F)** The bar chart shows the relative mRNAs levels of Nox-4 and p22phox in the parahippocampal cortex and hippocampus. **(G)** The bar chart shows the relative mRNAs levels of Nrf-2 and Ho-1 in the parahippocampal cortex and hippocampus. The results are expressed as the mean ± SD (*p < 0.05 vs. Normal; **p < 0.01 vs. Normal; ^#^*p* < 0.05 vs. SE; ^##^*p* < 0.01 vs. SE).

The present study showed that AST intervention significantly reduced oxidative stress status in SE rats and enhanced antioxidant activity *via* the Nrf-2/ARE pathway. Figures 6A,B showed that MDA in the plasma of the SE group-significantly increased compared to the Normal group (*p* < 0.05), and the SOD level decreased (*p* < 0.05). AST downregulated MDA (*p* < 0.05) level and upregulated SOD level (*p* < 0.05) in the plasma of SE rats. These results suggested that AST reduces the systemic oxidative stress response and enhances antioxidant capacity in SE rats. To evaluate the level of oxidative stress in the brain, we assayed the expression of oxidant indicators including p22phox and NADPH oxidase-4 (Nox-4) in the hippocampus and parahippocampal cortex of rats. Nox-4 is an NADPH oxidase, and p22phox is a subunit of NADPH oxidase. Both enzymes are major sources of reactive oxygen species (ROS). We detected the levels of protein and mRNA using western blot and RT-PCR, respectively. As shown in Figures 6C,D,F, the levels of nox-4 and p22phox protein and mRNA in the hippocampus and parahippocampal cortex of the SE group significantly increased compared to the Normal group, and AST reduced these changes (*p < 0.05 vs. Normal; **p < 0.01 vs. Normal; ^#^*p* < 0.05 vs. SE; ^##^*p* < 0.01 vs. SE). The levels of Nrf-2, heme oxygenase 1 (Ho-1) and sod1, which are major molecules of the antioxidant Nrf-2/ARE pathway, were detected using western blot and RT-PCR. The results showed that the levels of Nrf-2, Ho-1 and sod1 protein and mRNA in the hippocampus and parahippocampal cortex of rats in the SE group significantly decreased compared to the Normal group (*p < 0.05 vs. Normal; **p < 0.01 vs. Normal), and AST significantly elevated the expression of Nrf-2, Ho-1 and sod1 (^#^*p* < 0.05 vs. SE; ^##^*p* < 0.01 vs. SE; Figures 6C,E,G).

### Result 5. Astaxanthin Alleviated the Inflammatory Reaction in SE Rats *via* the NF-κB Pathway

**Figure 7:**
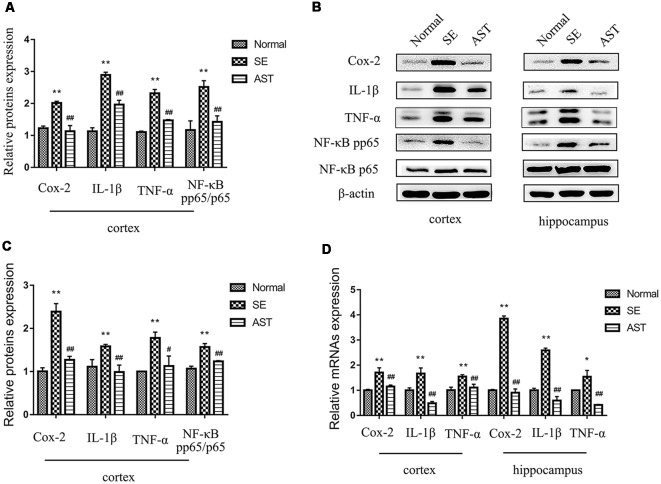
The inflammation levels in the parahippocampal cortex and hippocampus. **(A)** The bar chart shows the relative proteins levels of cyclooxygenase-2 (Cox-2), interleukin-1β (IL-1β), tumor necrosis factor-α (TNF-α) and NF-κB phosphorylation p65 (p-p65)/p65 in the parahippocampal cortex, compared with that of β-actin. **(B)** Representative protein bands of Cox-2, IL-1β, TNF-α, NF-κB p-p65 and p65 in hippocampus and the parahippocampal cortex. **(C)** The bar chart shows the relative proteins levels of Cox-2, IL-1β, TNF-α and NF-κB p-p65/p65 in the parahippocampus, compared with that of β-actin. **(D)** The bar chart shows the relative mRNAs levels of Cox-2, IL-1β and TNF-α in the parahippocampal cortex and hippocampus. The results are expressed as the mean ± SD (*p < 0.05 vs. Normal; **p < 0.01 vs. Normal; ^#^*p* < 0.05 vs. SE; ^##^*p* < 0.01 vs. SE).

The results indicated that AST intervention obviously alleviated the inflammatory response in the brains of SE rats, likely *via* the NF-κB pathway. The present study assayed the neuroinflammatory reaction by detecting the expression of classical inflammatory factors, including cyclooxygenase-2 (Cox-2), interleukin-1β (IL-1β) and tumor necrosis factor-α (TNF-α), in the hippocampus and parahippocampal cortex of rats using western blot and RT-PCR. As shown in Figure 7, the protein and mRNA levels of Cox-2, IL-1β and TNF-α in the hippocampus and parahippocampal cortex of the SE group significantly increased compared to the Normal group (*p < 0.05 vs. Normal; **p < 0.01 vs. Normal). However, AST intervention significantly reduced the expression levels of these inflammatory factors compared to the SE group (^#^*p* < 0.05 vs. SE; ^##^*p* < 0.01 vs. SE). To investigate the effects of AST on the NF-κB pathway, we also detected the levels of NF-κB p65 and phosphorylation p65 (p-p65) using western blot. The results showed that the expression of p-p65/p65 in the brain of rats in the SE group significantly increased compared to the Normal group (*p < 0.05 vs. Normal; **p < 0.01 vs. Normal), and AST reduced the expression of p-p65/p65 (^#^*p* < 0.05 vs. SE; ^##^*p* < 0.01 vs. SE; Figure 7).

### Result 6. Astaxanthin Reduced Neuronal Apoptosis in SE Rats *via* the PI3K/Akt Pathway

**Figure 8:**
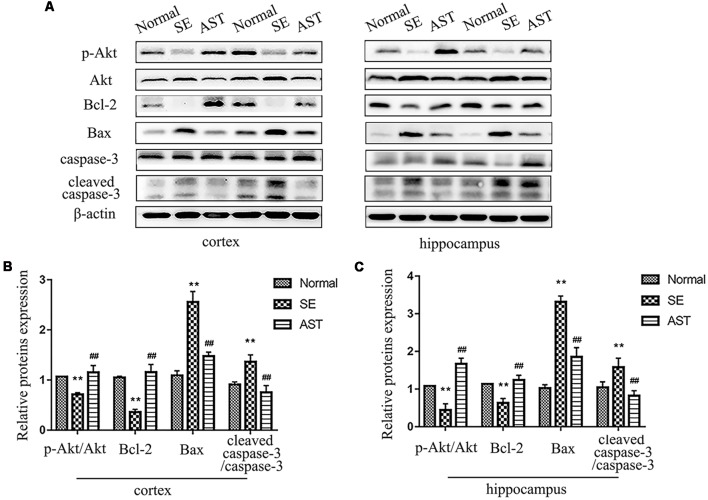
The expression of apoptosis regulation by the PI3K/Akt pathway. **(A)** Representative protein bands of p-Akt, Akt, B-cell lymphoma-2 (Bcl-2), Bax, caspase-3 and cleaved caspase-3 in the parahippocampal cortex and hippocampus. **(B)** The bar chart shows the relative proteins levels of p-Akt/Akt, Bcl-2, Bax and caspase-3/cleaved caspase-3 in the parahippocampal cortex, compared with that of β-actin. **(C)** The bar chart shows the relative proteins levels of p-Akt/Akt, Bcl-2, Bax and caspase-3/cleaved caspase-3 in the parahippocampus. The results are expressed as the mean ± SD (**p < 0.01 vs. Normal; ^##^*p* < 0.01 vs. SE).

The results suggested that AST alleviated SE-induced neuronal apoptosis *via* the PI3K/Akt pathway. TUNEL staining indicated that AST alleviated SE-induced neuronal apoptosis (Figure 5). We detected the expression of caspase-3 and cleaved caspase-3 in the hippocampus and parahippocampal cortex using western blot. The levels of cleaved caspase-3/caspase-3 in the hippocampus and parahippocampal cortex increased in the SE group compared to the Normal group (*p* < 0.01), which suggests the activation of apoptosis. AST reduced the expression of these proteins (*p* < 0.01). We detected the levels of molecules associated with the PI3K/Akt pathway using western blotting. B-cell lymphoma-2 (Bcl-2) associated X protein (bax) levels in the SE group increased and the levels of phospho-Akt/Akt and Bcl-2 decreased compared to the Normal group (**p < 0.01 vs. Normal). AST reduced Bax expression and increased the expression of p-Akt/Akt and Bcl-2 (^##^*p* < 0.01 vs. SE; Figure 8).

## Discussion

Early pathophysiological insults of SE in rodents include imbalanced oxidative stress, intense neuroinflammatory responses, selective neuronal degeneration, and disruption of the brain-blood barrier, which result in subsequent cognitive impairment (Walker, 2018). The longer the epileptic seizure lasts, the worse is the prognosis of neuron injury and cognitive function injury after SE (Trinka et al., 2016). The main therapeutic strategy of SE is termination of the epileptic seizure as soon as possible including first-line treatment (benzodiazepines) and second-line treatment (AEDs, including phenobarbital, valproic acid and levetiracetam). Some scholars insisted intravenous injections of midazolam, propofol and other anesthetics as soon as possible to terminate seizures (lasting for 30–60 min) rather than the use of multiple second-line drugs when the first-line treatment does not terminate the seizure (Betjemann and Lowenstein, 2015; Glauser et al., 2016). These drugs have obvious effects on the termination of seizures, but they do not effectively improve the pathophysiological changes after epilepsy or prevent SE from developing into chronic TLE. They even aggravate neuron damage or cognitive impairment (Agarwal et al., 2011; Sutter et al., 2014). Therefore, there is a pressing need for the development of new treatment strategies to alleviate the pathophysiological insults in the brain post-SE. We hypothesized that AST would exert neuroprotective effects in SE rats based on its effects on oxidative stress, inflammatory reactions and apoptosis.

Cognitive dysfunction is a common clinical manifestation of SE-induced brain injury. Long-term epileptic seizures inevitably affect the cognitive function of patients with SE and compromise the quality of life of these patients (Power et al., 2018). Previous studies reported that SE model rats using pilocarpine showed cognitive impairment compared to normal rats. Repeated transient seizures may lead to long-term deficits in spatial memory because of progressive hippocampal neuron loss induced by the repeated brief seizures (Kotloski et al., 2002; Müller et al., 2009). Our results also confirmed these results. AST treatment apparently improved the potential of learning and memory of SE rats. An increasing body of preclinical evidence over the past decade has demonstrated the neuroprotective effects of AST in multiple neurological diseases. A recent randomized, double-blind, placebo-controlled, small-sample clinical trial also showed that the addition of a compound containing AST and sesamin significantly improved cognitive function in patients with mild cognitive impairment (Ito et al., 2018).

Numerous studies demonstrated that inhibition of oxidative stress induced by seizure was enormously valuable. Activated oxidative stress in epilepsy and other neurological diseases accelerated chronic neuronal dysfunction and neurodegeneration. Oxidative stress activated by epileptic seizure may be a key incentive for recurrent seizures and exacerbate the consequences of seizures, such as neuronal death and cognitive dysfunction, and oxidative stress itself also results in neuronal death (Pearson-Smith and Patel, 2017). Elevated levels of oxidized proteins and lipids in the serum of patients with epilepsy indicated that oxidative stress was an ongoing process in epilepsy (Menon et al., 2012). Oxidative damage of biomolecules was detected in surgically resected epileptic brain tissue, and the oxidative stress reaction contributed to excitatory neural toxicity and neuronal degeneration (Pecorelli et al., 2015). Previous animal experiments demonstrated that antioxidant treatment exerted obvious neuroprotective effects in various epileptic models by alleviating oxidative stress in the brain (Pearson et al., 2015; Pauletti et al., 2017). Some treatments also improved neuronal injury after epilepsy by enhancing the Nrf2/ARE pathway to heighten the antioxidative activity of the body (Liu et al., 2018; Shi et al., 2018). The level of MDA in plasma increased in the SE group compared to the Normal group in the present study, the level of SOD in plasma decreased, and the levels of Nrf-2, Ho-1 and sod1 in the hippocampus and parahippocampal cortex decreased. However, AST reduced these changes. NADPH oxidase aggravated oxidative stress damage by inducing ROS (Brandes et al., 2014). NOX-4 is an important NADPH oxidative enzyme, and p22phox is a subunit of NADPH oxidase; both are vital biomarkers of oxidative stress. Therefore, we detected the levels of Nox-4 and p22phox in the brain. The results showed that SE increased the expression of Nox-4 and p22phox in the brain, and AST treatment decreased their expression. Previous studies demonstrated that the powerful antioxidant AST alleviated oxidative stress and enhanced anti-oxidative activity in other diseases (Ravi Kumar et al., 2016; Chalyk et al., 2017), which is consistent with our results. Therefore, our results suggest that AST plays a neuroprotective role in pilocarpine-induced injury *via* reducing oxidative stress and enhancing anti-oxidant capacity.

Burgeoning evidence from recent research studies considerably highlighted the key role of the inflammatory response in the pathogenesis of epilepsy. Inflammatory responses promote the occurrence and development of epilepsy. The brain tissue of patients with epilepsy undergoing surgical resection exhibits a strong inflammatory response, including reactive astrocyte proliferation, the infiltration of activated microglia, and the upregulation of various pro-inflammatory factors, including Cox-2, IL- 1β, TNF-α, and IL-6. Inflammatory cells, including mononuclear cells and neutrophil granulocytes, also increase in the brain, and inflammatory cells infiltrate from the periphery, which can promote inflammatory reactions in the brain and aggravate local neuronal injury (Varvel et al., 2016). Various SE model experiments also showed that rapid and persistent neuroinflammatory responses existed in the forebrain, which were mainly caused by activated microglia and astrocytes and infiltrated mononuclear cells. The inflammatory mediators induced by epileptic seizure include cytokines and their receptors. Inflammatory factors, such as Cox-2, IL-1β, and TNF-α, are rapidly induced in major anterior brain neurons such as hippocampal pyramidal cells and dentate granulosa cells. These increased pro-inflammatory factors are demonstrated to aggravate the proliferation of neuroglial cells, damage to the blood-brain barrier, neuronal excitability and seizure intensity, which may contribute to neuronal loss (Vezzani et al., 2015). Activation of the NF-κB pathway has been demonstrated to play a vital role in the process. There is a growing body of evidence derived from *in vitro* experiments and animal models that inhibition of the inflammatory response provides a significant neuroprotective effect and reduces the seizure degree and mortality (Serrano et al., 2011; Noe et al., 2013; Vezzani et al., 2015). Cox-2, IL-1β and TNF-α significantly increased in SE rats in the present study, and AST reduced these levels. AST also inhibited the activation of NF-κB. Numerous studies demonstrated that the application of AST reduced the expression of inflammatory factors such as IL-1β and TNF-α, and AST may inhibit the inflammatory response *via* multiple pathways (Kim et al., 2010, 2017; Ying et al., 2015). Our results suggest that AST suppresses the inflammatory reaction in pilocarpine-induced SE *via* the NF-κB pathway.

SE resulting in neurodegeneration, especially hippocampal neurons, is a prominent feature of clinical epilepsy (Dam, 1980). SE contributes to neuronal damage, which results in multiple complications, including cognitive impairment. The longer the seizure lasts, the more serious are the consequences. Oxidative stress and the inflammatory reaction vitally affect epilepsy-induced neuronal death. However, the underlying molecular mechanisms are yet completely understood. Previous studies reported that the neuronal death in the brain after SE mainly includes apoptosis and necrosis, and apoptosis is an important factor (Yu et al., 2018). Our study showed that neuronal apoptosis in the hippocampal CA1 region was obvious in SE rats using TUNEL staining, which is consistent with previous studies (Kotloski et al., 2002). TUNEL staining directly detects apoptotic cells, and the Bcl-2 protein family regulates apoptosis by controlling the release of mitochondrial apoptosis factors, cytochrome c, and apoptosis induction factors, which activate downstream executive reactions, including caspases (Zamzami and Kroemer, 2001). Previous studies demonstrated that caspase-3 was significantly activated in the brain of rats with epilepsy, cleaved caspase-3 significantly increased, the pro-apoptotic protein Bax increased, and the anti-apoptotic protein Bcl-2 decreased (Akcali et al., 2005; Yu et al., 2018). Our results confirmed these changes, and AST reversed these changes. A variety of signaling pathways regulate apoptosis. The PI3K/Akt pathway is a classical signal transduction pathway with various biological effects, and it exerts multiple roles in the regulation of cell growth, proliferation, differentiation and survival. The PI3K/Akt signaling pathway regulates apoptosis *via* regulating the expression of apoptosis-related molecules (Jin et al., 2017). The PI3K/Akt pathway plays an important role in epilepsy-induced neuronal damage, and activation of the PI3K/Akt pathway exhibits neuroprotective effects in epilepsy (Xue et al., 2011), which is consistent with our results. Therefore, we suggest that AST reduces SE-induced neuron damage *via* the PI3K/Akt pathway.

In brief, the present study demonstrated that AST exerted significant neuroprotective effects on neurological damage after SE. The data highlighted that AST afforded neuroprotection after a delayed post-injury intervention. AST blocked the rapid onset oxidative stress, inflammatory pathway and neuronal death during SE. AST, or co-treatment with AEDs, is likely to be a promising strategy to improve treatment efficacy.

## Data Availability

All datasets generated for this study are included in the manuscript.

## Author Contributions

XD performed the western blot and RT-PCR, generated the figures, and wrote the manuscript. SH and YS performed MRI, H&E staining and TUNEL staining of hippocampus. YF, YX and MWu established the SE models and performed EEG. MWa and XD performed the Morris water maze experiments. YC and XS contributed to the design and performance of experiments and revised the manuscript.

## Conflict of Interest Statement

The authors declare that the research was conducted in the absence of any commercial or financial relationships that could be construed as a potential conflict of interest.

## Abbreviations

SE: status epilepticus
AEDs: anti-epileptic drugs
TLE: temporal lobe epilepsy
AST: astaxanthin
Nrf-2: NF-E2-related factor 2
ARE: antioxidant response element
MRI: magnetic resonance imaging
HE: hematoxylin-eosin
TUNEL: TdT-mediated dUTP Nick-End Labeling
RT-PCR: real-time Polymerase Chain Reaction
Nox-4: NADPH oxidase-4
Ho-1: heme oxygenase 1
ROS: Reactive Oxygen Species
Cox-2: cyclooxygenase-2
IL-1β: interleukin-1β
TNF-α: tumor necrosis factor-α
bax: B-cell lymphoma-2
Bcl-2: associated X protein Bcl-2.
